# Systematic reviews of epidemiology in diabetes: finding the evidence

**DOI:** 10.1186/1471-2288-5-2

**Published:** 2005-01-08

**Authors:** Pamela Royle, Lynda Bain, Norman Waugh

**Affiliations:** 1 Department of Public Health, University of Aberdeen, Foresterhill, Aberdeen AB25 2ZD, Scotland

## Abstract

**Background:**

Methodological research to support searching for those doing systematic reviews of epidemiological studies is a relatively neglected area. Our aim was to determine how many databases it is necessary to search to ensure a comprehensive coverage of the literature in diabetes epidemiology, with the aim of examining the efficiency of searching in support of systematic reviews of the epidemiology of diabetes

**Methods:**

Three approaches were used. First, we defined a set of English language diabetes journals and examined their coverage in bibliographic databases. Second, we searched extensively for diabetes epidemiology articles (in all languages) to determine which are the most useful databases; and third, we analysed the scattering of these articles to determine the core journals in the area.

**Results:**

The overlap between MEDLINE and Embase for diabetes journals was 59%. A search for diabetes epidemiology articles across both MEDLINE and Embase, showed that MEDLINE alone retrieved about 94% of the total articles. Searching for diabetes epidemiology studies beyond MEDLINE and Embase retrieved no additional English language journal articles. The only diabetes epidemiology studies found by searching beyond MEDLINE and Embase were found in LILACS, and were Spanish or Portuguese language studies from Latin America; no additional English language studies were found. Only 30% of the meeting abstracts were converted to full publication after three years. One third of journal articles were published in just six journals, with *Diabetes Care *contributing 14.3% of the articles, followed by *Diabetic Medicine *(5.0%); *Diabetes Research & Clinical Practice *(4.1%); *Diabetologia *(4.0%); *Diabetes & Metabolism *(2.4%) and *Diabetes *(2.0%).

**Conclusions:**

Our results show that when searching for articles on diabetes epidemiology, MEDLINE and Embase would suffice for English language papers, with LILACS giving some additional non-English articles from Latin America. Although a MEDLINE-only search will retrieve the vast majority of the relevant literature, Embase and LILACs should also be searched to ensure the search is comprehensive. Searching for meeting abstracts is recommended to alert reviewers to unpublished work. The low rate of full publication of meeting abstracts has the danger of producing bias in reviews. Our findings on scattering show that the core literature in this field is concentrated in just six journals.

## Background

Review articles have a valuable role in the medical literature, because the volume of journals and articles is such that keeping up to date is very difficult. Reviews are much more valuable if they are systematic reviews done to internationally agreed standards, as non-systematic reviews are known to be subject to bias [1,2].

Dickersin noted that there was a shortage of systematic reviews in epidemiology, and called for more reviews, and more research into the methodology relating to reviews in epidemiology [3]. A study by Breslow found that more than 60% of epidemiology reviews were not methodologically systematic [4], and there has been little methodological research relating to their performance. Also, the methods have not been standardised, and the literature searching has not been supported, as done by the Cochrane Collaboration to support systematic reviews of clinical trials.

One of the key quality criteria for systematic reviews is the comprehensiveness of the searching, as failure to identify all relevant reports can result in selection bias[5]. The usual first source for identifying studies for reviews is MEDLINE, which currently indexes 4780 titles [6] of the estimated 14 000 biomedical titles currently published throughout the world [7]. In addition to searching MEDLINE, it is recommended that an extensive range of additional sources are searched [1].

A study, using *Ulrich's International Periodicals Directory *as a gold standard, found that a MEDLINE search in psychiatry would retrieve only about half the relevant journals [8]. Similarly, we were interested to investigate the coverage of diabetes journals and diabetes epidemiology articles in medical databases, in order to determine how many databases it is necessary to search to ensure a comprehensive coverage of the literature in diabetes epidemiology.

The scattering of the journal literature in a subject area can provide a useful insight into the number and range of journals needed to capture the key literature in a field. Bradford's Law of Scattering states that on any one subject, a small group of 'core' journals (Zone 1) will provide about one third of the articles on that subject, a medium number of less-core journals (Zone 2) will provide another third, and a large number of peripheral journals (Zone 3) will provide the final third of the articles [9,10].

The aim of this study was to examine the efficiency of searching in support of systematic reviews of the incidence and prevalence of diabetes by providing empirical data to answer the following questions:

1. Which databases index diabetes journals (restricted to English language)?

2. Which databases outside MEDLINE and Embase index diabetes epidemiology journal articles and grey literature?

3. How are diabetes epidemiology articles scattered across the journals, and what are the core journals in this area?

Accordingly, this study was divided into three parts.

(Note: This study is concerned only with searching for the epidemiology of diabetes itself, not with its complications).

## Methods

For the purposes of this study, epidemiology articles were defined as studies of incidence or prevalence of diabetes, or of factors affecting those (thereby excluding studies looking at other epidemiological aspects such as mortality). Basic science studies, e.g. biological mechanisms of disease, were not included.

We started from a position that both MEDLINE and Embase should always both be searched, since the overlap between these databases has been estimated to range from 10% to 87% [5,11-17]. Also, it is recognised that many relevant studies will appear in non-diabetes journals, and in sources not indexed in MEDLINE or Embase.

Hence, a three-part approach was used to investigate literature searching to support systematic reviews of diabetes epidemiology, and address each of our aims in turn.

### Part one: defining a set of 'diabetes' journals, and determining the databases in which theses journal were indexed

Diabetes journals were identified from the 'Medical Sciences – Endocrinology' section of *Ulrich's Periodical Directory *2003 (a comprehensive bibliographic database providing detailed information on periodicals published throughout the world) [18]. This was supplemented with a search of PubList.com [19] for journals with 'diabet' in title.

The inclusion criteria for the journals were: i) the word stem 'diabet' in the title, ii) contains original scientific studies of an academic or scholarly nature, iii) currently in print, and iv) published in English. If inclusion could not be decided on the basis of the information provided in *Ulrich's*, the contents pages for the past five years, and where necessary, abstracts or the full journal articles, were examined by a diabetes epidemiologist.

Journals which fulfilled all the above criteria were then checked against the *List of Serials Indexed for Online Users *[6] to see if they were indexed in MEDLINE. To determine if a journal was indexed by Embase, a search was done of 'diabet$' in the Journal Word (JW) field using the Embase OVID search interface, and limiting to publication year 2003.

If any journals were not indexed in either MEDLINE or Embase, searches of BIOSIS, BNI, CINAHL, SCI were done to determine if the journals were indexed in any of these databases.

### Part two: searching databases other than MEDLINE and Embase for journal articles and grey literature on diabetes epidemiology

Databases searched were: AMED, ASSIA, BIOSIS (abstracts only), BNI, CINAHL, Conference Papers Index, Dissertation Abstracts US, Health Management Information Consortium (HMIC), Index to Theses UK, ISI Proceedings, PsycINFO, NLM Gateway, LILACS (Latin American and Caribbean Health Sciences), National Research Register (NRR), SIGLE, SCI (abstracts only), SSCI, and Zetoc.

The search strategy used was 'diabetes and (epidemiology or incidence or prevalence)' in the Title (TI) field and restricted to publication years 1998 to 2003.

The titles (and abstracts when available) of all records were checked by an expert in diabetes epidemiology, in order to determine their potential usefulness for those doing systematic reviews.

### Part three: investigating the scatter of diabetes epidemiology journal articles found in a search of MEDLINE and Embase, and determining the core journals in this area

MEDLINE and Embase were searched using the search strategy: 'Diabetes Mellitus as the major subject heading, with the sub-heading 'Epidemiology' assigned (which includes incidence and prevalence), and restricted to publication years 1998 to 2003'. All languages were included. Duplicates found in both MEDLINE and Embase were removed. The journal titles in which the articles were found were ranked according to the number of articles contributed by each journal. The cumulated numbers of articles and journals were calculated and plotted. This was used to identify Bradford zones; that is, the number of journals needed to cover about one third, two thirds or all the relevant articles in the field.

## Results

### Part one: defining a set of 'diabetes' journals, and determining the databases in which theses journal were indexed

Searches of *Ulrich's Periodicals Directory *and *PubList.com *initially identified four English language journals that were of potential interest but not indexed by MEDLINE or Embase. On closer inspection, three of these, *Clinical Diabetes, Journal of Diabetes Nursing*, and *Diabetes and Primary Care *were excluded as they did not appear to contain any primary research; the articles were mainly educational, professional news and views, opinions, or narrative reviews. The fourth journal, *The Diabetic Foot*, contained primary research, but did not appear to contain studies useful for epidemiological reviews of diabetes itself (as opposed to complications). It is indexed by CINAHL only.

As shown in Table 1, 27 English language diabetes were covered collectively by MEDLINE and Embase in 2003. Seventy-four percent (20) were indexed in MEDLINE, 85% (23) in Embase, and 59% (16) were in both MEDLINE and Embase.

**Table 1:** English language diabetes journals indexed in either MEDLINE or Embase in 2003

	**Indexed in**
Acta Diabetologica	MEDLINE & Embase
Diabetes	MEDLINE & Embase
Diabetes & Metabolism	MEDLINE & Embase
Diabetes Care	MEDLINE & Embase
Diabetes Research	MEDLINE & Embase
Diabetes Research & linical Practice	MEDLINE & Embase
Diabetes Technology & Therapeutics	MEDLINE & Embase
Diabetes, Nutrition & Metabolism – Clinical & Experimental	MEDLINE & Embase
Diabetes, Obesity & Metabolism	MEDLINE & Embase
Diabetes/Metabolism Research Reviews.	MEDLINE & Embase
Diabetic Medicine	MEDLINE & Embase
Diabetologia	MEDLINE & Embase
Experimental & Clinical Endocrinology & Diabetes	MEDLINE & Embase
Experimental Diabesity Research	MEDLINE & Embase
Journal of Diabetes & its Complications	MEDLINE & Embase
Pediatric Diabetes	MEDLINE & Embase
Current Diabetes Reports	MEDLINE
Diabetes Educator	MEDLINE
Diabetes Forecast	MEDLINE
Diabetes Self-Management	MEDLINE
British Journal of Diabetes & Vascular Disease	Embase
Canadian Journal of Diabetes	Embase
Cme Bulletin Endocrinology & Diabetes.	Embase
Current Opinion in Endocrinology & Diabetes	Embase
Diabetologia Croatica	Embase
Journal of Endocrinology, Metabolism & Diabetes of South Africa	Embase
Practical Diabetes International.	Embase

The four diabetes journals unique to MEDLINE were all published in the USA. By contrast, only one of the seven journals unique to Embase was published in the USA.

### Part two results: searching databases other than MEDLINE and Embase for journal articles and grey literature on diabetes epidemiology

The results are summarised below:

#### Journal articles (English language)

No English language journals articles that were not also indexed in MEDLINE or Embase were identified.

#### Journal articles (non-English language)

There were 23 Spanish and Portuguese language articles identified in LILACs. On the basis of the English translation of the titles, they all reported studies done in Latin America.

#### Grey literature

We defined grey literature as any literature not published in a peer reviewed journal. After removing duplicates, there were 51 dissertations identified from searches of Dissertations Abstracts US, Index to Theses UK, and SIGLE. The research presented in the vast majority (92%) of the dissertations appeared to have been written up as articles in journals indexed in MEDLINE or Embase. No grey literature studies of any format other than dissertations were retrieved from SIGLE, so there was very little additional information gained by these searches.

#### Research in progress

The National Research Register (a database of ongoing and recently completed research projects funded by, or of interest to, the United Kingdom's National Health Service) gave brief details of 18 projects in progress that had not been otherwise identified. Searching the NRR might be useful if unpublished results could be included in the review, but its main value would be to indicate when the review was likely to need updating.

#### Meeting abstracts and conference proceedings

The search of the Conference Proceedings Index retrieved 25 articles, none of which appeared to have been published as journal articles after five years. The Zetoc Conference Search found eight articles, of which 50% had been published as full journal articles in MEDLINE or Embase.

The search of Science Citation Index (SCI), restricted to meeting abstracts only, found 171 relevant studies. The time to publication of the SCI abstracts was examined by checking how many had subsequently been published as journal articles indexed in MEDLINE or Embase. It was found that 30% had reached full publication after three years.

A search of BIOSIS, restricted to meeting abstracts only, retrieved 71 additional relevant abstracts that were not in SCI. Most (65%) of these 71 abstracts came from the supplements of *Diabetes and Metabolism *and *Diabetes Research and Clinical Practice*. Of these, 11 (12%) had been published in journals. The average time delay from the date of publication of the abstract to full publication was 1.4 years.

#### Databases searched where no articles not in MEDLINE or Embase were found

These included AMED, BNI, HMIC, NLM Gateway meeting abstracts, PsychINFO, and SSCI,

In summary, the data indicate that when searching for English language journal articles on diabetes epidemiology, searches of MEDLINE and Embase would suffice. The exception would be for studies from Latin America, where LILACS should also be searched. Searching for meeting abstracts may alert reviewers to forthcoming or unpublished work.

### Part three: investigation of the scatter of diabetes epidemiology journal articles found in a search of MEDLINE and Embase, and determination of the core journals in this area

The searches for diabetes epidemiology articles in MEDLINE and Embase resulted in 2923 articles being found in 696 different journal titles; 39% were found to be in 'diabetes journals' and 14% were non-English language.

**Figure 1:**
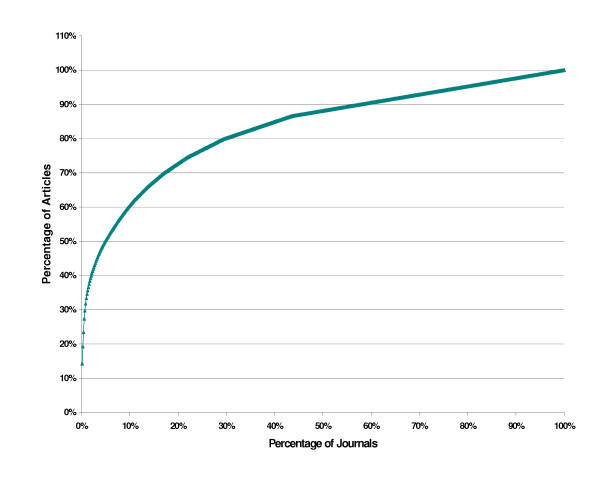
Distribution of diabetes epidemiology articles across journals

Figure 1 shows the distribution of all 696 articles retrieved across the journals.

Applying Bradford's Law of Scattering gives three zones, each providing one-third of the articles.

#### Zone 1

The first one-third of articles were from six journals. They are in rank order: *Diabetes Care *(contained 14.3% of the articles); *Diabetic Medicine *(5.0%); *Diabetes Research & Clinical Practice *(4.1%); *Diabetologia *(4.0%); *Diabetes & Metabolism *(2.4%), *Diabetes *(2.0%) These six journals represent 0.9% (of the 696) total journals, and all are indexed in MEDLINE.

#### Zone 2

The second one-third of articles were from 62 journals, representing 9.1% of the total journals. The four journals in Embase only were: *Practical Diabetes International*; *Diabetologia Polska*; *Diabetes und Stoffwechsel *and *Journal of the Japan Diabetes Society*. Hence, 94% of Zone 2 journals are covered by MEDLINE.

#### Zone 3

The final one-third of articles were in 628 journals, representing 90.2% of the total journals. MEDLINE indexed 88% of these journals.

Overall, for the three zones, the search of MEDLINE and Embase for diabetes epidemiology articles revealed that MEDLINE indexed 89% of the total journals, and these contained 94% of the articles.

## Discussion

Our results showed that there was an overlap of only 59% in current English language 'diabetes journals' indexed by both MEDLINE and Embase. Also, a search for diabetes epidemiology articles across both MEDLINE and Embase showed that MEDLINE alone retrieved about 94% of the total articles; therefore, both databases should be searched. Embase appears to index more diabetes journals published outside the USA. Therefore, if searching is limited to MEDLINE only (Embase being less accessible and more expensive than MEDLINE, which is free via PubMED) this could potentially introduce a bias. Also, duplication of searching can be useful, as due to differences in indexing practices, a search of one database may retrieve something missed by the other.

We also found that despite a wide range of additional databases searched after MEDLINE and Embase, no additional English language journal articles on diabetes epidemiology were identified. The LILACS database was a useful source of Spanish and Portuguese language articles on the epidemiology of diabetes in Latin American countries.

Meeting abstracts appeared to be valuable sources of information on forthcoming studies, but their inclusion in systematic reviews is contentious. Some reviewers exclude abstracts on the grounds that the quality of the study cannot be judged because of the inevitably limited detail. However, others include them on the grounds that abstracts provide the most up-to-date information.

Nearly all the dissertations identified had been published as journal articles. However, it was found that only 30% of the meeting abstracts were converted to full publication after three years, which is considerably lower than the figure for RCTs, which is 56% [20]. This has the danger of producing bias in systematic reviews, if failure to publish is based on the size and direction of study results.

Scattering of the diabetes epidemiology articles revealed that the 'core' literature in this field is concentrated in just six journals, with *Diabetes Care *alone containing about 14% of the articles. A similar concentration effect in journals was also shown in a study of 3400 science journals in the SCI database, where just 100 journals accounted for 22% of the published articles and 100 journals also accounted for 44% of cited articles [21].

This study has a number of limitations. The search to identify diabetes journals was restricted to English language journals only, as we were unable to assess articles in other languages. We did not compare the quality of the articles identified from databases outside MEDLINE and Embase. Also, when searching for articles, we were necessarily limited to the range of databases available to us.

Finally, there may be databases inaccessible or unknown to us that cover foreign language and regional journals not indexed in MEDLINE and Embase. Such journals may carry studies of incidence which may seem of primarily local interest, but which may be useful contributions to the international body of evidence because they may show large variations in incidence, or in its relationship to possible aetiological risk factors.

It is often useful to study the epidemiology of a disease where it is rare, as well as where it is common. However it is likely that studies which report high incidence are more likely to be published than those which report low incidence. Similarly with risk factors; a study which finds no link between factor x and disease y may be less likely to be published than one which does show a correlation [22,23].

There is a need for further research to see whether our findings apply to searching for epidemiological reviews of other diseases, and on measuring the sensitivity and specificity of various search filters to retrieve epidemiological studies in MEDLINE and Embase.

We endorse Dickersin's suggestion of an international collaborative effort to establish an 'epidemiological Cochrane-like database' to identify all relevant studies and to begin systematically reviewing available data for important epidemiological questions [3].

## Conclusions

Searching MEDLINE and Embase appears to provide comprehensive coverage of the English language journal literature in diabetes epidemiology. LILACs is a useful source of Spanish and Portuguese language articles on diabetes epidemiology done in Latin American countries and published in regional journals not indexed in MEDLINE and Embase. Searching for meeting abstracts is recommended to alert reviewers to unpublished work.

The volume of literature on diabetes epidemiology makes it impossible for one person to read everything. However the provision of systematic reviews makes keeping up with research manageable, and more reviews are needed. Our findings on scattering shows that the core literature in diabetes epidemiology is concentrated in a small number of core journals, and that in the absence of reviews, one can follow the field by reading these journals. It may also be reassuring that a good MEDLINE-only search will retrieve the vast majority of the relevant literature.

## Competing interests

The author(s) declare that they have no competing interests.

## Authors' contributions

PR and NW conceived the study and drafted the initial manuscript. PR and LB collected and analysed the data. All authors contributed to and approved the final manuscript.

## Pre-publication history

The pre-publication history for this paper can be accessed here:


